# The genetics of host–virus coevolution in invertebrates

**DOI:** 10.1016/j.coviro.2014.07.002

**Published:** 2014-10

**Authors:** Darren J Obbard, Gytis Dudas

**Affiliations:** 1 Institute of Evolutionary Biology, University of Edinburgh, Kings Buildings, Edinburgh, UK; 2 Centre for Infection Immunity and Evolution, University of Edinburgh, Kings Buildings, Edinburgh, UK

## Abstract

Although viral infection and antiviral defence are ubiquitous, genetic data are currently unavailable from the vast majority of animal phyla — potentially biasing our overall perspective of the coevolutionary process. Rapid adaptive evolution is seen in some insect antiviral genes, consistent with invertebrate-virus ‘arms-race’ coevolution, but equivalent signatures of selection are hard to detect in viruses. We find that, despite the large differences in vertebrate, invertebrate, and plant immune responses, comparison of viral evolution fails to identify any difference among these hosts in the impact of positive selection. The best evidence for invertebrate-virus coevolution is currently provided by large-effect polymorphisms for host resistance and/or viral evasion, as these often appear to have arisen and spread recently, and can be favoured by virus-mediated selection.

## Highlights

• No genetic data are available on viruses or antiviral defence for most animal phyla.
• The research bias may bias understanding of host–virus coevolution.
• Antiviral RNAi genes of Drosophila display rapid adaptive evolution.
• There is no difference between plant, vertebrate, and insect viruses in median *dN*/*dS* ratio.
• High-frequency large-effect segregating polymorphisms provide evidence for coevolution.

**Current Opinion in Virology** 2014, **8**:73–78

This review comes from a themed issue on **Virus evolution**

Edited by **Michael Brockhurst** and **Scott Hensley**

For a complete overview see the Issue and the Editorial

Available online 24th July 2014

**http://dx.doi.org/10.1016/j.coviro.2014.07.002**

1879-6257/© 2014 The Authors. Published by Elsevier Ltd. This is an open access article under the CC BY license (http://creativecommons.org/licenses/by/3.0/).

## Background

Viral infection and antiviral defence are universal phenomena [1] and viral infections are reported across the metazoa [2, 3, 4]. However, research tends to focus more on the coevolution of vertebrates (and plants) and their viruses than on invertebrates and their viruses, and relevant genetic data on viruses and antiviral resistance are lacking for almost all invertebrate phyla. If major lineages differ systematically in their molecular or ecological interaction with viruses, as might be expected given the differences in immune mechanisms, then the research bias could skew our overall perspective of host–virus (co)evolutionary process [e.g. 5^•^].

In this review we present data from arthropods that broadly suggest viruses do indeed drive invertebrate evolution — selective sweeps, resistance polymorphisms, and elevated rates of protein evolution have all been attributed to virus-mediated selection. However, whether this is part of a strict coevolutionary process [6, 7] is less clear: viruses certainly evolve in response to invertebrate hosts, but as yet there is relatively little evidence demonstrating that this occurs as part of a reciprocal selective process.

## Virus-driven invertebrate evolution

Selection by viruses could drive frequent and rapid fixations in invertebrate populations, reducing genetic diversity at the selected loci and elevating divergence between species. Selection on amino-acid sequences, which may be common for antagonistic host–virus interaction, could additionally elevate the rate of non-synonymous substitution (*dN*). Comparison of such ‘footprints of selection’ between immune genes and genes with other functions argues in favour of pathogen-mediated selection in arthropods generally [8, 9, 10, 11], and identifies the antiviral RNAi pathway as a potential coevolutionary hotspot in *Drosophila* [9, 12•, 13]. Genes mediating antiviral RNAi [Ago2 and Dcr2, reviewed in 14] are among the fastest evolving 3% of protein sequences across *D. melanogaster* and *D. simulans*, with adaptive amino-acid fixations in this pathway estimated to happen every 10–40 thousand years [15]. Moreover, there is evidence for positive selection and recent selective sweeps in antiviral RNAi genes from multiple *Drosophila* lineages, while homologous ‘housekeeping’ genes do not show this pattern [12•, 15, 16].

The hypothesis that this is driven by a molecular ‘arms race’ with viruses is appealing [15], first because virus-encoded suppressors of RNAi (VSRs) are widespread among RNA viruses [reviewed in 17], second because some VSRs are known to interact directly with AGO2 and DCR2 [18, 19, 20], and third because VSRs from Drosophila Nora viruses can be highly specific to the host species’ AGO2 [21^••^]. However, other invertebrate antiviral genes are not reported to display extensive positive selection, and it remains possible that selection on *Drosophila* RNAi genes has been mediated by other selective agents [22]. To test whether such potential ‘hot spots’ of immune system evolution are a general phenomenon will require data from a wider range of invertebrate taxa, and based on sequence analysis alone it will remain hard to attribute selection to the action of viruses.

Virus-mediated selection may also be inferred using high-frequency large-effect host resistance polymorphisms, as these can result from negative frequency dependent selection (i.e. when rare alleles have higher fitness) or incomplete/ongoing selective sweeps [reviewed in 7]. A large-effect polymorphism in the *D. melanogaster* autophagy-pathway gene *ref(2)P* conveys resistance to the vertically-transmitted *Drosophila melanogaster* Sigma Virus (DMelSV), with the resistant allele reducing viral transmission by ∼90% in females and ∼60% in males [reviewed in 23]. The resistant allele occurs at 25–35% in European populations, and population-genetic analyses suggest it arose roughly 1–10Kya and has increased in frequency recently [24, 25]. A second large-effect DMelSV resistance polymorphism comprises a natural *Doc* transposable element insertion into *CHKov1* followed by a partial duplication and inversion involving *CHKov1* and *CHKov2*. The *Doc* insertion exists at high frequency (80% in a North American population) and reduces infection rates by ∼50%. The subsequent rearrangement gave rise to a virus-inducible *CHKov2* transcript associated with an 80–140 fold decrease in viral titre [26]. Again, population genetic analyses of this locus suggest resistance is derived and has recently increased in frequency [26, 27]. Resistance to Drosophila C virus (DCV) is associated with segregating variants in *pastrel* (∼50% increase in survival time) and *Anaphase promoting complex 7* (>100% increase, but this currently lacks experimental verification [28^••^]), although both resistant alleles are currently rare [15% and 3% of surveyed alleles in the wild, see 28^••^]. Finally, experimental evolution under recurrent challenge with DCV also identified functional polymorphism in *pastrel*, and further identified virus-resistant alleles segregating in *Ubc-E2H* and *CG8492*. The DCV-resistant alleles of *pastrel* and *Ubc-E2H* respectively displayed a 24% and 14% selective advantage under experimental conditions, and knock-downs of gene expression reduced survival after challenge [29^••^].

High-frequency large-effect viral resistance polymorphisms have also been reported from other invertebrates. For example, segregating resistance to the Orsay Virus in the nematode *Caenorhabditis elegans* maps to a non-functional truncation of *Drh-1*, one of three dicer-related helicases involved in RNAi [30^•^]. Here the susceptible allele is derived, but is nevertheless found at a global frequency of 23% and appears to have spread recently, perhaps suggesting the action of selection at a linked locus [30^•^]. Polymorphism in the antiviral RNAi pathway (*Dicer-2*) has also been proposed to underlie some of the genetic variance for resistance to Dengue virus in the mosquito *Aedes aegypti* [31]. In other cases the mechanism for resistance is unknown. For example, some populations of the pest moth *Cydia pomonella* have recently evolved resistance to its Granulosis virus, via a single dominant sex-linked allele that blocks viral replication [32, 33]. Similarly, resistance to White Spot Syndrome Virus in the shrimp *Penaeus monodon* has been mapped to single marker associated with a *∼*2000-fold reduction in viral titre [34], which occurs at a frequency of 40–60% [35].

Such polymorphisms are consistent with negative frequency dependent selection or with incomplete/ongoing selective sweeps [e.g. 28^••^], but because the resistant allele is often recently derived and increasing in frequency, it seems likely that many may be in the process of fixing. However, robustly attributing evolution to virus-mediated selection is challenging, and selection by other agents [e.g. *Doc* insertion in *CHKov1*; 27], and at linked loci [e.g. *drh-1* deletion; 30^•^] have been proposed in some cases. Nevertheless, experimental evolution shows that virus-mediated selection can lead to a rapid evolutionary response in *Drosophila* and can select for segregating variants such as *pastrel* [29^••^] and *ref(2)P* [36].

## Invertebrate-driven virus evolution

It seems certain that viral evolution occurs in response to invertebrates, if only because hosts always dominate the viral environment. For example, viral adaptation may underlie host-specificity seen in some insect viruses [21••, 37, 38], and adaptation to the invertebrate host has been attributed to specific amino-acid changes in several invertebrate-vectored viruses, including Chikungunya Virus, Venezuelan equine encephalitis virus, and West Nile Virus [39, 40, 41]. Such adaptation to the host may also be reflected by the tendency for Sigma Viruses to replicate more effectively in closer relatives of their natural hosts [42].

Given this, it is interesting to ask whether virus evolution occurs in response to specific host immune mechanisms. Genotype by genotype interactions — with host polymorphism for resistance and viral polymorphism for overcoming that resistance — may be indicative of negative frequency-dependent selection or incomplete on-going selective sweeps in the virus, driven by selection mediated by host resistance. For example, genotype by genotype interactions have been reported between Dengue Virus 1 and *Aedes aegypti* mosquitoes [43, 44]. The best-studied invertebrate case may be the interaction between *ref(2)P* and DMelSV [23, 45], where a viral lineage capable of overcoming *ref(2)P* resistance arose a few hundred years ago and subsequently spread to become the most common form [46, 47]. The rapid spread of this resistance-insensitive virus was documented as it occurred in two European populations [48, 49], and experiments suggest that the *ref(2)P*-insensitive virus can replace the sensitive virus in a resistant *ref(2)P* host background — indicating that host resistance may indeed drive viral evolution [36]. The rapid spread of a viral lineage may often be indicative of a selective sweep, and such expansions have also been seen in the Sigma virus of *D. obscura* [50]. However, without additional evidence of pre-sweep genotypes or genomic regions such potential sweeps cannot be differentiated from expansions [e.g. an epidemic, 51], and cannot be attributed to host-mediated selection.

It is often argued that if host resistance drives the recurrent appearance of novel viral protein variants, then this may elevate the ratio of non-synonymous to synonymous variants (*dN*/*dS*) in the virus [52, 53]. This is widely accepted for some viral genes interacting with the vertebrate immune system [52, 54], but although several multi-isolate invertebrate datasets are available [46, 47, 50, 55, 56, 57, 58, 59, 60, 61, 62, 63, 64], few present whole genomes or analyse patterns of protein evolution [c.f. 51]. However, some vertebrate and plant viruses interact with their invertebrate vectors, allowing the additional impact of invertebrate-mediated selection over and above that mediated by vertebrates or plants to be detected [65, 66]. Previous analyses of viral surface proteins — which often interact directly with host proteins — suggests that *dN*/*dS* is lower in vector-borne viruses [*dN*/*dS* = 0.07 vs 0.17 for vertebrates, 0.10 vs 0.19 for plants; see 65, 66], either because of increased constraint imposed by alternating selective environments, or because of reduced positive selection.

**Figure 1:**
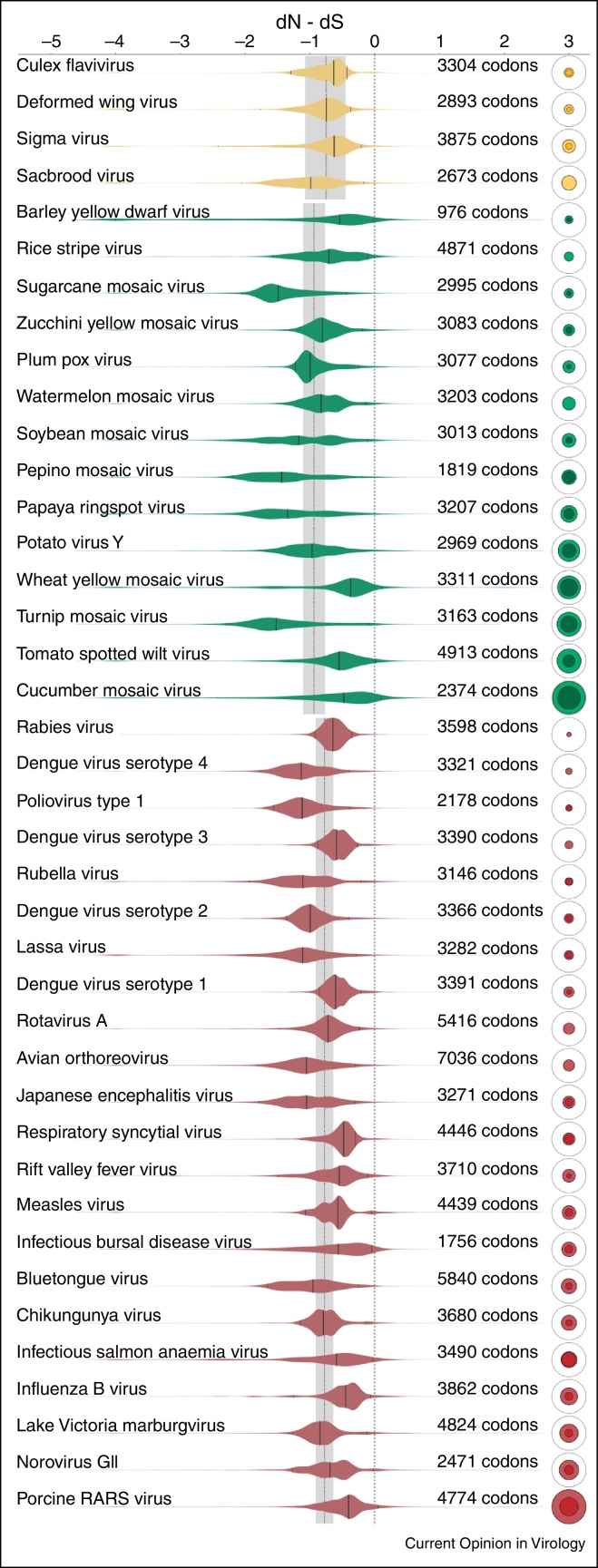
Constraint and positive selection in the protein-coding sequences of 40 RNA viruses infecting vertebrates, invertebrates, or plants. Plots illustrate the distribution of estimated *dN-dS* for all codons in the complete coding sequence of each virus (insects yellow, plants green and vertebrates red; median and 95th percentiles are marked; *dN* = *dS* implies neutrality). The *dN-dS* summary statistic is used in place of *dN/dS* because estimates are more stable and tend to be closer to Gaussian in their distribution. Grey boxes indicate the 95% credible interval for each category mean, estimated using a Generalised Linear Mixed Model (GLMM). Coloured circles indicate the number of positively selected codons (PSCs), that is, those estimated to have *dN* > *dS* at a posterior probability of 0.8 (pale circles: max = 55 min = 1) or 0.9 (dark circles: max = 25 min = 0). A GLMM found no significant difference between host types in the median viral *dN–dS* (likely to reflect overall constraint) or in the number of PSCs (likely to reflect the impact of positive selection). Note that the number of PSCs did not correlate with the total number of codons. Viruses were chosen to encompass a wide phylogenetic distribution, and were included if ≥20 complete genomes were available (≥16 complete genomes for invertebrates). If >100 genomes were available, the data were down-sampled at random to 100 sequences. Selection was inferred using FUBAR [71] from the HyPhy package [72] on a 20 × 20 grid with 10 independent MCMC chains each providing 1000 subsamples from the posterior (each 5 × 10^8^ steps after 5 × 10^8^ burn-in steps). Codons were only included if the effective sample size from the posterior was ≥100. Overlapping reading frames were excluded and recombination breakpoints were inferred using GARD [73] before FUBAR analysis. GLMMs were fitted using MCMCglmm [74], with host as a fixed effect and viral family as a random effect. A Gaussian distribution was assumed for median *dN–dS* values, while the number of PSCs was assumed to be Poisson distributed. Significance was assessed by examination of the credibility intervals.

It was suggested that vector-borne vertebrate viruses may display reduced *dN/dS* partly because the impact of positive selection (detected as sites with *dN* > *dS*) is reduced; first, because fewer viruses tested ‘positive’ for adaptive evolution [1 of 17 vs 12 of 27; 65], and second, because the difference in *dN*/*dS* between vectored and non-vectored viruses was reduced when putatively positively selected sites were excluded [*dN*/*dS* = 0.13 vs 0.06; 65]. Interestingly, the only viruses in which positive selection was often detectable were non-vectored vertebrate viruses [65, 66]. Taken together, these data may suggest that constraint is higher in vector-borne viruses, but that neither plants nor invertebrates are as likely as vertebrates to drive viral *dN* detectably above *dS*. Figure 1 presents a new analysis for 40 complete RNA virus genomes [55, 56], sampled broadly across plant and animal hosts. We were unable to identify any systematic difference between the viruses of plants, insects and vertebrates in either the median *dN–dS* value or the number of positively selected codons. However, while invertebrate viruses are not strikingly different from the others, the extremely small sample size (*n* = 4) precludes any firm conclusions regarding patterns of viral protein evolution in invertebrate hosts.

## Conclusions

Despite the evidence for strong positive selection acting on some antiviral immunity genes, there are generally few sites in the viruses of vertebrates, arthropods, or plants which exhibit detectable positive selection using the *dN* > *dS* test, and the number does not differ significantly between these groups (Figure 1). There is generally little evidence for pervasive diversifying selection in either surface proteins [65, 66] or VSRs [67]. However, even assuming that *dN* > *dS* is a good metric of positive selection, there are at least two reasons why it may be hard to detect an arms race using such data from RNA viruses. First, if hosts drive global selective sweeps to fixation in the virus, then standing *dN/dS* within a population will not strongly reflect the impact of positive selection [53]. Second, even if different viral lineages respond in parallel to selection — so that comparisons between the lineages might be expected to display elevated *dN*/*dS* — the disparity in evolutionary rates means that host fixations will be so infrequent, compared to viral mutations, as to have virtually no impact on viral *dN/dS* [e.g. 67]. Therefore it is perhaps unsurprising that the well-known examples of pervasive diversifying selection in viruses are not driven by coevolution with the host population, but by virus evolution in response to the rapidly changing ‘adaptive’ immune response of vertebrates [e.g. 54].

Given the difficulty associated with inferring invertebrate-virus coevolution from historic patterns of protein evolution, the best evidence instead comes from patterns of functional polymorphism. Although the most compelling case is arguably the *ref(2)P*-DMelSV system, in which resistance and the ability to overcome it have both arisen recently and increased in frequency, and each is known to be selectable by the other [reviewed in 23], such large-effect polymorphisms increasingly appear common in invertebrate-virus interaction. This mirrors what is seen for plant–virus interaction [68] and some other invertebrate–pathogen systems [e.g. 69], where large-effect host polymorphisms for resistance and/or virus polymorphisms for evasion or suppression seem almost universal [i.e. ‘gene-for-gene’ and ‘matching alleles’ models; see 70], and suggest that ongoing and/or incomplete sweeps may be widespread. Indeed, if viral insensitivity to resistance often arises rapidly, before the resistant allele has fixed, then reciprocal invertebrate–virus coevolution may be much more widespread than is evident from reciprocal sweeps to fixation.

## References and recommended reading

Papers of particular interest, published within the period of review, have been highlighted as:

• of special interest
•• of outstanding interest

## References

[1] Koonin E.V., Dolja V.V. (2013). A virocentric perspective on the evolution of life. Curr Opin Virol.

[2] Johnson P.T. (1984). Viral diseases of marine invertebrates. Helgolander Meeresuntersuchungen.

[3] Renault T., Novoa B. (2004). Viruses infecting bivalve molluscs. Aquatic Living Res.

[4] Williams T. (2008). Natural invertebrate hosts of iridoviruses (Iridoviridae). Neotrop Entomol.

[5•] Desbiez C., Moury B., Lecoq H. (2011). The hallmarks of “green” viruses: do plant viruses evolve differently from the others?. Infect Genet Evol.

[6] Janzen D.H. (1980). When is it coevolution?. Evolution.

[7] Woolhouse M.E.J., Webster J.P., Domingo E., Charlesworth B., Levin B.R. (2002). Biological and biomedical implications of the co-evolution of pathogens and their hosts. Nat Genet.

[8] Sackton T.B., Lazzaro B.P., Schlenke T.A., Evans J.D., Hultmark D., Clark A.G. (2007). Dynamic evolution of the innate immune system in *Drosophila*. Nat Gen.

[9] Obbard D.J., Welch J.J., Kim K.-W., Jiggins F.M. (2009). Quantifying adaptive evolution in the *Drosophila* immune system. PLoS Genet.

[10] McTaggart S.J., Obbard D.J., Conlon C., Little T.J. (2012). Immune genes undergo more adaptive evolution than non-immune system genes in *Daphnia pulex*. BMC Evol Biol.

[11] Roux J., Privman E., Moretti S., Daub J.T., Robinson-Rechavi M., Keller L. (2014). Patterns of positive selection in seven ant genomes. Mol Biol Evol.

[12•] Kolaczkowski B., Hupalo D.N., Kern A.D. (2011). Recurrent adaptation in RNA interference genes across the *Drosophila* phylogeny. Mol Biol Evol.

[13] Nolte V., Pandey R.V., Kofler R., Schlotterer C. (2013). Genome-wide patterns of natural variation reveal strong selective sweeps and ongoing genomic conflict in *Drosophila mauritiana*. Genome Res.

[14] Nayak A., Tassetto M., Kunitomi M., Andino R., Cullen B.R. (2013). Intrinsic Immunity.

[15] Obbard D.J., Jiggins F.M., Halligan D.L., Little T.J. (2006). Natural selection drives extremely rapid evolution in antiviral RNAi genes. Curr Biol.

[16] Obbard D.J., Jiggins F.M., Bradshaw N.J., Little T.J. (2011). Recent and recurrent selective sweeps of the antiviral RNAi gene argonaute-2 in three species of *Drosophila*. Mol Biol Evol.

[17] Li F., Ding S.-W. (2006). Virus counterdefense: diverse strategies for evading the RNA-silencing immunity. Annu Rev Microbiol.

[18] van Mierlo J.T., Bronkhorst A.W., Overheul G.J., Sadanandan S.A., Ekström J.-O., Heestermans M., Hultmark D., Antoniewski C., van Rij R.P. (2012). Convergent evolution of argonaute-2 slicer antagonism in two distinct insect RNA viruses. PLoS Pathog.

[19] Nayak A., Berry B., Tassetto M., Kunitomi M., Acevedo A., Deng C., Krutchinsky A., Gross J., Antoniewski C., Andino R. (2010). Cricket paralysis virus antagonizes Argonaute 2 to modulate antiviral defense in *Drosophila*. Nat Struct Mol Biol.

[20] Qi N., Zhang L., Qiu Y., Wang Z., Si J., Liu Y., Xiang X., Xie J., Qin C.-F., Zhou X. (2012). Targeting of dicer-2 and RNA by a viral RNA silencing suppressor in Drosophila cells. J Virol.

[21••] van Mierlo J.T., Overheul G.J., Obadia B., van Cleef K.W.R., Webster C.L., Saleh M.-C., Obbard D.J., van Rij R.P. (2014). Novel *Drosophila* viruses encode host-specific suppressors of RNAi. PLoS Pathog.

[22] Obbard D.J., Gordon K.H.J., Buck A.H., Jiggins F.M. (2009). The evolution of RNAi as a defence against viruses and transposable elements. Philos Trans R Soc B-Biol Sci.

[23] Longdon B., Wilfert L., Jiggins F.M., Dietzgen R. (2012). Rhabdoviruses: Molecular Taxonomy, Evolution, Genomics, Ecology, Cytopathology and Control.

[24] Wayne M.L., Contamine D., Kreitman M. (1996). Molecular population genetics of *ref(2)P*, a locus which confers viral resistance in *Drosophila*. Mol Biol Evol.

[25] Bangham J., Obbard D.J., Kim K.W., Haddrill P.R., Jiggins F.M. (2007). The age and evolution of an antiviral resistance mutation in *Drosophila melanogaster*. Proc Roy Soc B-Biol Sci.

[26] Magwire M.M., Bayer F., Webster C.L., Cao C.A., Jiggins F.M. (2011). Successive increases in the resistance of Drosophila to viral infection through a transposon insertion followed by a duplication. PLoS Genet.

[27] Aminetzach Y.T., Macpherson J.M., Petrov D.A. (2005). Pesticide resistance via transposition-mediated adaptive gene truncation in Drosophila. Science.

[28••] Magwire M.M., Fabian D.K., Schweyen H., Cao C., Longdon B., Bayer F., Jiggins F.M. (2012). Genome-wide association studies reveal a simple genetic basis of resistance to naturally coevolving viruses in *Drosophila melanogaster*. PLoS Genet.

[29••] Martins N.E., Faria V.G., Nolte V., Schlötterer C., Teixeira L., Sucena É., Magalhães S. (2014). Host adaptation to viruses relies on few genes with different cross-resistance properties. Proc Natl Acad Sci.

[30•] Ashe A., Belicard T., Le Pen J., Sarkies P., Frezal L., Lehrbach N.J., Felix M.A., Miska E.A. (2013). A deletion polymorphism in the *Caenorhabditis elegans* RIG-I homolog disables viral RNA dicing and antiviral immunity. Elife.

[31] Lambrechts L., Quillery E., Noël V., Richardson J.H., Jarman R.G., Scott T.W., Chevillon C. (2013). Specificity of resistance to dengue virus isolates is associated with genotypes of the mosquito antiviral gene Dicer-2. Proc Roy Soc B Biol Sci.

[32] Asser-Kaiser S., Fritsch E., Undorf-Spahn K., Kienzle J., Eberle K.E., Gund N.A., Reineke A., Zebitz C.P.W., Heckel D.G., Huber J. (2007). Rapid emergence of baculovirus resistance in codling moth due to dominant, sex-linked inheritance. Science.

[33] Asser-Kaiser S., Radtke P., El-Salamouny S., Winstanley D., Jehle J.A. (2011). Baculovirus resistance in codling moth (*Cydia pomonella* L.) caused by early block of virus replication. Virology.

[34] Dutta S., Biswas S., Mukherjee K., Chakrabarty U., Mallik A., Mandal N. (2014). Identification of RAPD-SCAR marker linked to white spot syndrome virus resistance in populations of giant black tiger shrimp, *Penaeus monodon* Fabricius. J Fish Dis.

[35] Chakrabarty U., Mallik A., Mondal D., Dutta S., Mandal N. (2014). Assessment of WSSV prevalence and distribution of disease-resistant shrimp among the wild population of *Penaeus monodon* along the west coast of India. J Invertebrate Pathol.

[36] Fleuriet A. (1982). Factors affecting the frequency of infection by the Sigma virus is experimental populations of *Drosophila melanogaster*. Arch Virol.

[37] Cory J.S., Myers J.H. (2003). The ecology and evolution of insect baculoviruses. Annu Rev Ecol Evol Syst.

[38] Wijkamp I., Almarza N., Goldbach R., Peters D. (1995). Distinct levels of specificity in thrips transmission of Tospoviruses. Phytopathology.

[39] Moudy R.M., Meola M.A., Morin L.L.L., Ebel G.D., Kramer L.D. (2007). A newly emergent genotype of west Nile virus is transmitted earlier and more efficiently by Culex mosquitoes. Am J Trop Med Hyg.

[40] Brault A.C., Powers A.M., Ortiz D., Estrada-Franco J.G., Navarro-Lopez R., Weaver S.C. (2004). Venezuelan equine encephalitis emergence: Enhanced vector infection from a single amino acid substitution in the envelope glycoprotein. Proc Natl Acad Sci U S A.

[41] Tsetsarkin K.A., Weaver S.C. (2011). Sequential adaptive mutations enhance efficient vector switching by chikungunya virus and its epidemic emergence. PLoS Pathogens.

[42] Longdon B., Hadfield J.D., Webster C.L., Obbard D.J., Jiggins F.M. (2011). Host phylogeny determines viral persistence and replication in novel hosts. PLoS Pathogens.

[43] Lambrechts L., Chevillon C., Albright R.G., Thaisomboonsuk B., Richardson J.H., Jarman R.G., Scott T.W. (2009). Genetic specificity and potential for local adaptation between dengue viruses and mosquito vectors. BMC Evol Biol.

[44] Fansiri T., Fontaine A., Diancourt L., Caro V., Thaisomboonsuk B., Richardson J.H., Jarman R.G., Ponlawat A., Lambrechts L. (2013). Genetic mapping of specific interactions between *Aedes aegypti* mosquitoes and dengue viruses. PLoS Genet.

[45] Wilfert L., Jiggins F.M. (2013). The dynamics of reciprocal selective sweeps of host resistance and a parasite counter-adaptation in *Drosophila*. Evolution.

[46] Carpenter J.A., Obbard D.J., Maside X., Jiggins F.M. (2007). The recent spread of a vertically transmitted virus through populations of *Drosophila melanogaster*. Mol Ecol.

[47] Wilfert L., Jiggins F.M. (2014). Flies on the move: an inherited virus mirrors *Drosophila melanogaster*'s elusive ecology and demography. Mol Ecol.

[48] Fleuriet A., Periquet G. (1993). Evolution of the *Drosophila melanogaster* Sigma virus system in natural populations from Languedoc (Souther France). Arch Virol.

[49] Fleuriet A., Sperlich D. (1992). Evolution of the *Drosophila melanogaster* Sigma virus system in natural population from Tubingen. Theor Appl Genet.

[50] Longdon B., Wilfert L., Obbard D.J., Jiggins F.M. (2011). Rhabdoviruses in two species of Drosophila: vertical transmission and a recent sweep. Genetics.

[51] Robles-Sikisaka R., Bohonak A.J., McClenaghan L.R., Dhar A.K. (2010). Genetic signature of rapid IHHNV (infectious hypodermal and hematopoietic necrosis virus) expansion in wild penaeus shrimp populations. PLoS ONE.

[52] Yang Z.H., Nielsen R., Goldman N., Pedersen A.M.K. (2000). Codon-substitution models for heterogeneous selection pressure at amino acid sites. Genetics.

[53] Kryazhimskiy S., Plotkin J.B. (2008). The population genetics of dN/dS. PLoS Genet.

[54] Tusche C., Steinbruck L., McHardy A.C. (2012). Detecting patches of protein sites of influenza A viruses under positive selection. Mol Biol Evol.

[55] Palacios G., Hui J., Quan P.L., Kalkstein A., Honkavuori K.S., Bussetti A.V., Conlan S., Evans J., Chen Y.P., vanEngelsdorp D. (2008). Genetic analysis of israel acute paralysis virus: distinct clusters are circulating in the United States. J Virol.

[56] Allen C., Briano J.A., Varone L., Oi D.H., Valles S.M. (2010). Exploitation of a high genomic mutation rate in *Solenopsis invicta* virus 1 to infer demographic information about its host, *Solenopsis invicta*. J Invertebrate Pathol.

[57] Wertheim J.O., Tang K.F.J., Navarro S.A., Lightner D.V. (2009). A quick fuse and the emergence of Taura syndrome virus. Virology.

[58] Baker A., Schroeder D. (2008). Occurrence and genetic analysis of picorna-like viruses infecting worker bees of *Apis mellifera* L. populations in Devon, South West England. J Invertebrate Pathol.

[59] Forsgren E., Miranda J., Isaksson M., Wei S., Fries I. (2009). Deformed wing virus associated with Tropilaelaps mercedesae infesting European honey bees (*Apis mellifera*). Exp Appl Acarol.

[60] Berényi O., Bakonyi T., Derakhshifar I., Köglberger H., Topolska G., Ritter W., Pechhacker H., Nowotny N. (2007). Phylogenetic analysis of deformed wing virus genotypes from diverse geographic origins indicates recent global distribution of the virus. Appl Environ Microbiol.

[61] Singh R., Levitt A.L., Rajotte E.G., Holmes E.C., Ostiguy N., vanEngelsdorp D., Lipkin W.I., dePamphilis C.W., Toth A.L., Cox-Foster D.L. (2010). RNA viruses in hymenopteran pollinators: evidence of inter-taxa virus transmission via pollen and potential impact on non-apis hymenopteran species. PLoS ONE.

[62] Kapun M., Nolte V., Flatt T., Schlötterer C. (2010). Host range and specificity of the Drosophila C virus. PLoS ONE.

[63] Kim D.Y., Guzman H., Bueno R., Dennett J.A., Auguste A.J., Carrington C.V.F., Popov V.L., Weaver S.C., Beasley D.W.C., Tesh R.B. (2009). Characterization of Culex Flavivirus (Flaviviridae) strains isolated from mosquitoes in the United States and Trinidad. Virology.

[64] Ryabov E.V., Wood G.R., Fannon J.M., Moore J.D., Bull J.C., Chandler D., Mead A., Burroughs N., Evans D.J. (2014). A virulent strain of deformed wing virus (DWV) of honeybees (*Apis mellifera*) prevails after varroa destructor-mediated, or in vitro, transmission. PLoS Pathog.

[65] Woelk C.H., Holmes E.C. (2002). Reduced positive selection in vector-borne RNA viruses. Mol Biol Evol.

[66] Chare E.R., Holmes E.C. (2004). Selection pressures in the capsid genes of plant RNA viruses reflect mode of transmission. J Gen Virol.

[67] Murray G.G., Kosakovsky Pond S.L., Obbard D.J. (2013). Suppressors of RNAi from plant viruses are subject to episodic positive selection. Proc Roy Soc B.

[68] Fraile A., Garcia-Arenal F., Carr J.P., Loebenstein G. (2010).

[69] Luijckx P., Fienberg H., Duneau D., Ebert D. (2013). A matching-allele model explains host resistance to parasites. Curr Biol.

[70] Dybdahl M.F., Jenkins C.E., Nuismer S.L. (2014). Identifying the molecular basis of host-parasite coevolution: merging models and mechanisms. Am Nat.

[71] Murrell B., Moola S., Mabona A., Weighill T., Sheward D., Kosakovsky Pond S.L., Scheffler K. (2013). FUBAR: a fast, unconstrained bayesian approximation for inferring selection. Mol Biol Evol.

[72] Kosakovsky Pond S.L., Frost S.D.W., Muse S.V. (2005). HyPhy: hypothesis testing using phylogenies. Bioinformatics.

[73] Kosakovsky Pond S.L., Posada D., Gravenor M.B., Woelk C.H., Frost S.D.W. (2006). GARD: a genetic algorithm for recombination detection. Bioinformatics.

[74] Hadfield J.D. (2010). MCMC methods for multi-response generalized linear mixed models: the MCMCglmm R package. J Statistical Software.

